# Dependence of the Nonlinear Photoacoustic Response of Gold Nanoparticles on the Heat-Transfer Process

**DOI:** 10.1021/acs.jpcc.1c09245

**Published:** 2022-01-31

**Authors:** Jian-Ping Sun, Ya-Tao Ren, Zi-Xuan Liu, Ming-Jian He, Bao-Hai Gao, Hong Qi

**Affiliations:** † School of Energy Science and Engineering, Harbin Institute of Technology, Harbin 150001, China; ‡ Key Laboratory of Aerospace Thermophysics, Ministry of Industry and Information Technology, Harbin 150001, China; § Faculty of Engineering, University of Nottingham, University Park, Nottingham NG7 2RD, U.K.

## Abstract

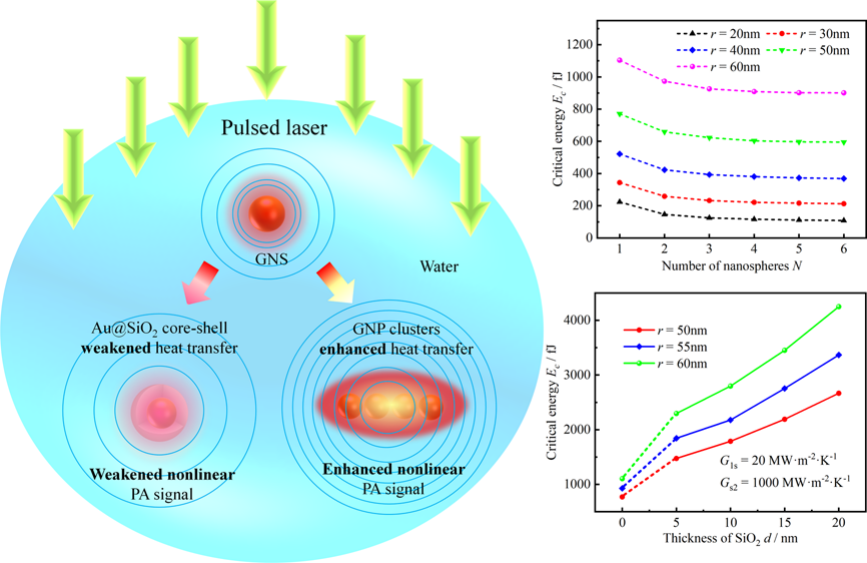

Photoacoustic (PA) imaging using the nonlinear PA response of gold nanoparticles (GNPs) can effectively attenuate the interference from background noise caused by biomolecules (e.g., hemoglobin), thus offering a highly potential noninvasive biomedical imaging method. However, the mechanism of the nonlinear PA response of GNPs based on the thermal expansion mechanism, especially the effect of heat-transfer ability, still lacks quantitative investigation. Therefore, this work investigated the effect of heat-transfer ability on the nonlinear PA response of GNPs using the critical energy and fluence concept, taking into account the Au@SiO_2_ core–shell nanoparticles (weakened heat transfer) and gold nanochains (enhanced heat transfer). The results showed that the stronger the heat transferability, the smaller the critical energy, indicating that the nonlinear PA response of different nanoparticles cannot be contrasted directly through the critical energy. Moreover, the critical fluence can directly contrast the proportion of nonlinear components in the PA response of different GNPs as governed by the combined effect of heat transferability and photothermal conversion ability.

## Introduction

Photoacoustic imaging (PAI) is a biomedical imaging method that combines the advantages of optical imaging and ultrasound imaging^1^ and has the characteristics of significant imaging depth, high resolution, noninvasion, and nonionization.^2^ Thus, PAI has attracted much interest in recent years as it offers excellent potential in medical diagnosis. PAI achieves functional imaging and medical diagnosis through the reconstruction of contrast agent distribution, which relies on the high optical absorption performance of the contrast agent. Noble metal plasmonic nanomaterials have huge optical absorption cross sections owing to localized surface plasmon resonance (LSPR).^3,4^ In particular, gold nanoparticles (GNPs) are ideal exogenous contrast agents for PAI due to their easy synthesis,^5,6^ absence of biological toxicity,^7^ and easy conjugation with targeting ligands.^8^ Hence, GNPs have been extensively studied and applied in the field of biomedical imaging.^9−18^

For biological tissue imaging, the pulsed laser should be below the critical dose to address biosafety concerns. In the present work, the excited PA response is the conversion of optical energy into thermal energy by GNPs, along with the linear thermal expansion with the surrounding water.^19^ This PA signal is closely related to the physical parameter of water, specifically the thermal expansion coefficient. When the thermal expansion coefficient of water becomes independent from the temperature change, the PA signal amplitude and the pulsed laser fluence become linear.^20^ However, when the temperature significantly changes, the correlation between the thermal expansion coefficient and temperature cannot be ignored. At this point, the PA signal amplitude and the pulsed laser fluence are approximately second-order related, so that the nonlinear PA response is excited.^21^

Quantitative reconstruction of the distribution of GNPs is required when utilizing the PA response for biomedical functional diagnosis. However, accurate reconstruction of the distribution of GNPs is challenging to achieve due to the intense background noise that can arise from endogenous biomolecules, such as hemoglobin. Furthermore, endogenous biomolecules have a low absorption cross section and do not generate a nonlinear PA response. By utilizing GNPs that excite nonlinear PA response, Schrof et al.^22^ reconstructed the distribution of gold nanospheres (GNSs) in the suspension using India ink as the background, representing the biomolecule hemoglobin. This finding implies that quantitative reconstruction of exogenous contrast agents in biological tissues utilizing the nonlinear PA response of GNPs is a feasible and promising strategy.

To develop quantitative photoacoustic (PA) tomography for biological tissues based on the nonlinear PA response of GNPs, one must characterize the mechanism of the nonlinear PA response of GNPs. Meanwhile, GNSs can maintain stable morphological characteristics at a relatively high pulse fluence, and the structure is simple and easy to synthesize. Hence, many mechanistic studies have focused on GNSs for nonlinear PA response. Given that radius is a core structural feature of GNSs, Prost et al.^23^ investigated the effect of GNS size following the study of Calasso et al.^24^ about the nonlinear PA response originating from a point heat source. The results showed that the nonlinear PA signal predicted by a point heat source was relatively large compared with the obtained GNSs. Moreover, Prost et al. proposed the concept of critical energy and critical fluence of GNPs to investigate the effects of GNSs radius, pulse duration, and reference temperature on the nonlinear PA response. Furthermore, Pang et al.^25^ and Simandoux et al.^26^ experimentally investigated the nonlinear PA response of GNS suspensions. They found that no significant nonlinear PA response was detected for 40 nm GNSs, suggesting that this may be a combined effect of the bandwidth limitation of the sonicator, the spatial distribution of GNSs, and GNS agglomeration. However, when the radius is above 50 nm, a remarkable nonlinear PA response can be detected under the irradiation of larger pulsed laser fluence.

GNSs require surface modification when they are used in practical applications. Very recently, Pang et al.^27^ found that GNSs with coated SiO_2_ would quench their nonlinear PA response, providing new possibilities for biosensors. Gandolfi et al.^28^ employed a numerical approach to study the dependence of the nonlinear PA response of GNSs in an aqueous solution on wavelength. They simultaneously considered the wavelength-dependent absorption coefficient of GNSs and water. The results showed that the nonlinear PA response of GNSs appeared at 532 nm (LSPR wavelength), whereas a strong linear PA signal (no nonlinear effect) could be detected at 750 nm in the near-infrared region (NIR). In contrast to GNSs with a small absorption coefficient in the NIR band, the PA signal for 750 nm excitation originated from water absorption for NIR light. Moreover, the in vitro study of Nam et al.^29^ found that GNSs with a radius of 20 nm are endocytosed by cells and undergo aggregation, which excite a nonlinear PA response. They suggested that this phenomenon resulted from a significant increase in the temperature of the medium surrounding the GNPs due to aggregation, altering the thermal expansion coefficient of water significantly. However, they did not conduct further theoretical studies.

**Figure 1:**
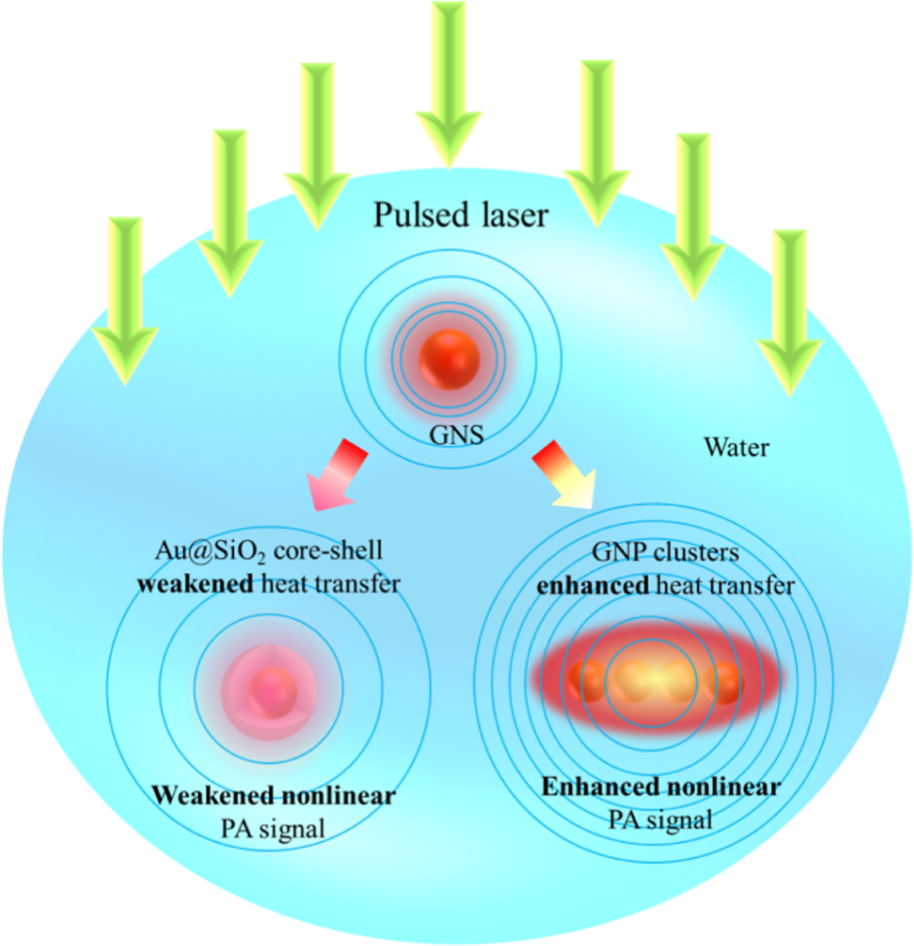
Schematic diagram of nonlinear PA response of heat transfer to GNPs.

The essence of the nonlinear PA response of GNSs based on the thermal expansion mechanism is that the temperature of water changes dramatically, resulting in a significantly large thermal expansion coefficient of water. Therefore, heat transfer has an important effect on exciting the PA response, as shown in Figure 1. However, there is currently a lack of quantitative studies on the impact of the heat-transfer process on the nonlinear PA response of GNSs. In this paper, we quantitatively investigate the concept of critical energy and critical fluence proposed by Prost et al.^23^ for the nonlinear PA response of GNSs, and the nonlinear PA response of Au@SiO_2_ core–shell nanoparticles (weakened heat transfer) and nanochains (enhanced heat transfer). Our study captures the aspect of heat transfer using the finite element method (FEM) to deepen the understanding of the nonlinear PA mechanism of GNPs.

## Methods

### PA Response Model of the GNP

When a linearly polarized plane wave illuminates the GNP with frequency ω, the electric field **E** and the magnetic field **H** can be calculated by Maxwell’s curl equations^30^
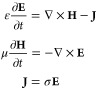
1where μ, ε, and σ are the relative permeability, relative permittivity, and conductivity, respectively, and **J** is the current density.

*C*_abs_ is the absorption cross section of the GNP, which can be calculated as^31^
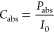
2where *I*_0_ is the incident laser fluence and *P* is the total energy absorbed or scattered by the nanoparticles. The expression of *P* is complex, which can be referred from ref (32).

The absorbed electromagnetic energy is converted into thermal energy of the GNP and can be calculated as the resistive heating *q*_r_(33)
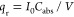
3where *V* is the volume of the nanoparticles.

The temperature of the GNP rises rapidly because of resistance heating. When illuminated by a nanosecond pulsed laser, the electronic temperature of the electronic gas is equal to the temperature of the lattice (phonons),^34^ which indicates that the heat transfer and temperature distribution in the GNP and surrounding water can be calculated by the transient Fourier heat conduction equation^35^
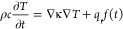
4where ρ, *c*, κ, and *T* are the density, heat capacity, thermal conductivity, and temperature, respectively. Here, the function *f*(*t*) represents the temporal Gaussian-shaped laser pulse, which is defined as
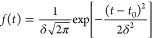
5where *t* is the time, *t*_0_ is the time position of the center of the peak, and  is the standard deviation. Here, *t*_w_ is the full width at half-maximum of the Gaussian profile defined as pulse duration.

There is interfacial thermal resistance at the interface of different material surfaces, making the temperature distribution on both sides of the interface discontinuous. The boundary conditions at the interface meet the following formula^36^
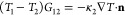
6where subscripts “1” and “2”, respectively, represent the media on both sides of the interface, *G* is the thermal conductivity of the interface, and **n** represents the normal direction of the interface.

The linear thermal expansion occurs due to an increased temperature in the GNP and the surrounding water. The corresponding stress–strain tensor can be calculated by the Duhamel–Hooke’s equation^37^
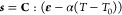
7where **s** is the total stress tensor, **C** is the fourth-order elasticity tensor related to shear and bulk modulus, “:” is defined as the double-dot product, **ε** is the total strain tensor, α is the thermal expansion coefficient, and *T*_0_ is the reference temperature.

The convection of fluid in the whole process of thermal expansion is ignored. Therefore, the water medium around the GNP can be regarded as a solid, and its elastic properties can be represented by shear and bulk moduli.^38^

The thermal expansion-induced total displacement **u** can be calculated from the total stress tensor **s** through the following equation^37^
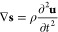
8

The acoustic pressure change can be calculated from the thermal expansion-induced total displacement **u** by^37^
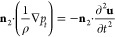
9where **n**_2_ is the outward normal to the boundary and *p*_t_ = *p* + *p*_b_ is the total acoustic pressure. Here, *p* is the PA signal and *p*_b_ is the ambient pressure.

The propagation and pressure distribution of the PA signal in the surrounding medium can be obtained by solving the following acoustic wave equation^20^
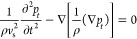
10where *v*_s_ is the speed of sound.

**Figure 2:**
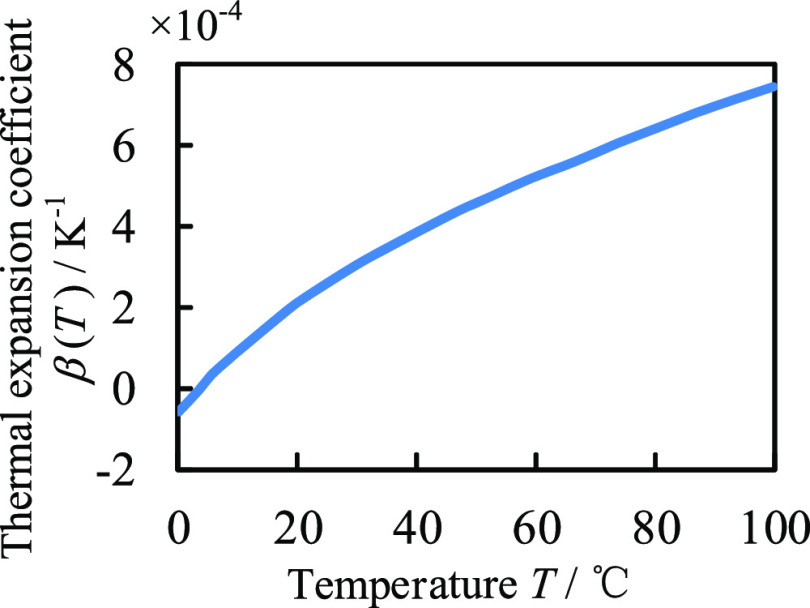
Thermal expansion coefficient of water as a function of temperature. Adapted with permission from ref (23). Copyright 2015 American Physical Society.

The physical parameters of Au, SiO_2_, and H_2_O are shown in Table 1 and Figure 2.

**Table 1:** Physical Parameters Used for the PA Model

parameters	symbol	value
density of gold	ρ_Au_	19,300 kg/m^3^
density of silica	ρ_SiO_2__	2320 kg/m^3^
density of water	ρ_H_2_O_	1000 kg/m^3^
thermal conductivity of gold	κ_Au_	318 W/m/K
thermal conductivity of silica	κ_SiO_2__	1.44 W/m/K
thermal conductivity of water	κ_H_2_O_	0.6 W/m/K
shear modulus of gold	*Y*_Au_	75 × 10^9^ N/m^2^
shear modulus of silica	*Y*_SiO_2__	71 × 10^9^ N/m^2^
Poisson’s ratio of gold	ν_Au_	0.42
Poisson’s ratio of silica	ν_SiO_2__	0.17
shear modulus of water	*G*_H_2_O_	0 N/m^2^
bulk modulus of water	*K*_H_2_O_	2.15 × 10^9^ N/m^2^
expansion coefficient of gold	β_Au_	0.42 × 10^–6^ 1/K
expansion coefficient of silica	β_SiO_2__	0.55 × 10^–6^ 1/K
expansion coefficient of water	β_H_2_O_	shown in Figure 2
heat capacity of gold	*c*_Au_	129 J/kg/K
heat capacity of silica	*c*_SiO_2__	740 J/kg/K
heat capacity of water	*c*_H_2_O_	4200 J/kg/K
sound velocity of water	*v*_s_H_2_O_	1500 m/s

### Simulation Methodology

The absorption cross section of gold nanorods is calculated by finite difference time domain (Lumerical). The dielectric function of gold is implemented into the calculations using the experimental results measured by Johnson and Christy,^39^ and the refractive index of SiO_2_ is implemented into the calculations using the results in ref (40). The refractive index of water is 1.33. The perfectly matched layer is used as the external boundary condition to eliminate the influence of reflected electromagnetic wave. In the calculation, there are dense grids near the GNPs. The number of grids in the narrow place (such as SiO_2_ shell and nanospheres’ gap) is not less than 5, which is adjusted according to the specific size of the narrow place.

The photothermal and PA responses of gold nanorods, including the coupled partial differential equations of transient heat-transfer equation, transient structural mechanics equation, and transient sound propagation, are solved by FEM (Comsol Multiphysics). The coupling variable between the Fourier heat conduction equation and the generalized Hooke’s law equation is temperature *T*. An isothermal wall, equal to the initial temperature 293.15 K, is used in the heat-transfer equation. Meanwhile, in the structural mechanics equation, both GNPs and water are linear thermal expansion media, and others are the default settings. The coupling variables of structural mechanics equation and sound propagation equation are displacement **u** and pressure *p*, which are coupled by eq 9. The boundary condition is the spherical wave radiation at the outermost boundary to reduce the influence of reflected sound waves in the sound propagation equation.

## Results and Discussion

**Figure 3:**
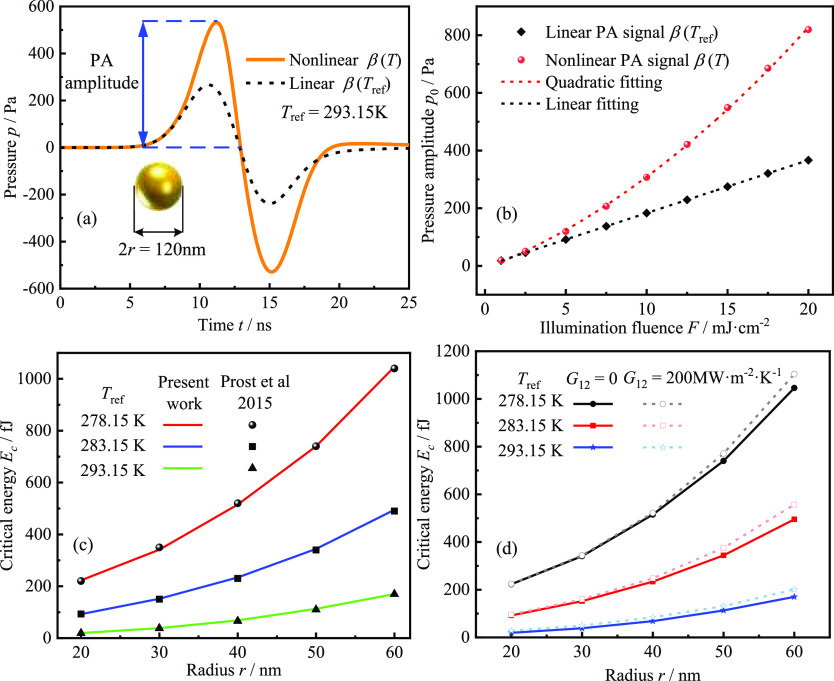
GNSs’ nonlinear PA signal and FEM validation: (a) temporal profiles of linear and nonlinear PA pressure (detection point at 750 nm from the center of the GNS) at a reference temperature of 293.15 K, a pulse duration of 5 ns, and absorption energy as the critical energy and (b) linear vs nonlinear PA signal as a function of laser fluence. The nanosphere radius, reference temperature, and pulse duration are the same as in (a); (c) critical energies *E*_c_ of Au nanospheres with different radii at different reference temperatures calculated by FEM are compared with the results in ref (23); (d) critical energies *E*_c_ of different GNS radii at different reference temperatures when considering interfacial thermal resistance vs not considering interfacial thermal resistance.

Under the irradiation of the nanosecond pulsed laser, the suspension of GNPs will produce a PA signal, that is, the initial PA pressure in the field of PAI. In relation to the Gruneisen parameter Γ = βc_s_^2^/*c*, absorption coefficient μ_a_, and the laser fluence , satisfy *p*_0_ = Γμ_a_*F*.^25^ However, during excitation of the PA signal, the temperature of the surrounding water rises rapidly, which results in an increased coefficient of thermal expansion (see Figure 2). As a result, the magnitude of the nanoparticle PA signal is no longer proportional to the pulsed laser fluence but exhibits nonlinearity, as shown in Figure 3a,b. To quantitatively investigate the nonlinear PA response of nanoparticles based on the thermal expansion mechanism, Prost et al.^23^ defined the critical energy *E*_c_ as the value of absorbed energy for which the peak amplitudes of the nonlinear and linear contributions in the detected PA signal were identical.

For GNPs, this work shows that the nonlinear contribution is defined by the difference between the total signal predicted in the nonlinear regime and the signal predicted in the linear regime only by keeping β constant, as shown in Figure 3a. To verify the accuracy of the model established in this paper in describing the nonlinear problems of GNPs, we calculate the critical energy *E*_c_ of GNSs with different radii at different reference temperatures (i.e., initial ambient temperatures) when the pulse duration is 5 ns. We further compare our findings with the results in ref (23), as shown in Figure 3c, revealing that our results are in good agreement with those in ref (23). However, ref (23) ignored the interfacial thermal resistance between gold and water, which is not optimal in practice. In fact, the nanoparticle nonlinear PA response based on the thermal expansion mechanism is caused by the temperature change of water. In contrast, the interfacial thermal resistance has a specific effect on the heat transfer between gold and water in the transient temperature response.

This work also considers the interfacial thermal resistance model in the heat-transfer process and investigates its effect on the critical energy, as shown in Figure 3d. The interfacial thermal resistance gives a temperature difference at the interface of GNSs and water,^41^ decreasing the heat-transfer ability between them; hence, the rise of water temperature decreases. To make the nonlinearity the same as the linear contribution, GNSs need to absorb greater thermal energy to bring the temperature elevation of water to the level where the interfacial thermal resistance is neglected. Therefore, Figure 3d shows that the presence of interfacial thermal resistance gives a weak increase in the critical energy *E*_c_.

**Figure 4:**
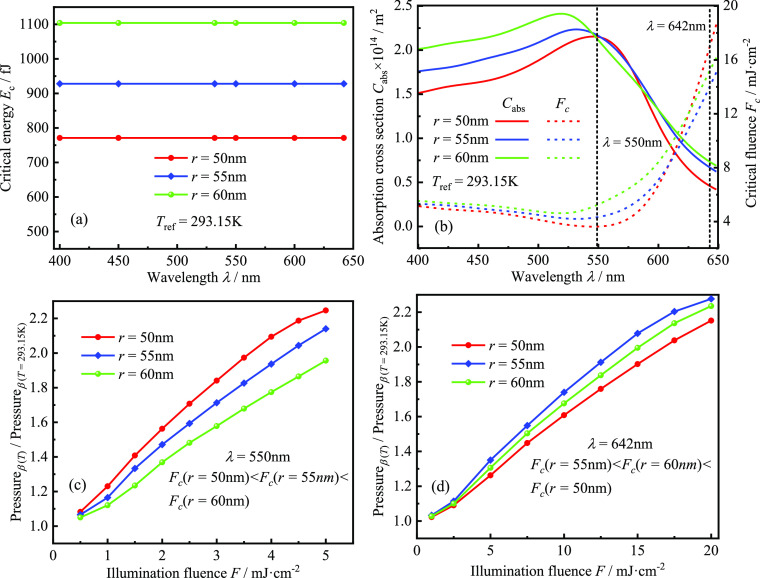
Effect of laser wavelength on the nonlinear PA response of GNSs (reference temperature 293.15 K and pulse duration 5 ns): (a) critical energy of GNSs with different radii as a function of laser wavelength; (b) absorption cross sections and critical laser fluence of GNSs with different radii as a function of laser wavelength; and (c,d) ratio of the total acoustic pressure amplitude to the linear acoustic pressure amplitude as a function of laser fluence at 550 and 642 nm, respectively.

The critical energy is helpful to study the nonlinear PA signal of GNPs quantitatively. Still, it is impossible to directly measure the strength of the nonlinear PA signal (the proportion of nonlinear components in the detected nonlinear PA signal) for different sizes or morphologies of GNPs. Moreover, the critical energy cannot directly illustrate the effect of the laser wavelength on the nonlinear PA signal of GNPs, as shown in Figure 4a. The result suggests that the critical energy is independent of the laser wavelength. Therefore, the critical laser fluence *F*_c_ is the laser fluence of pulsed laser light when the nonlinear contribution in the PA signal of GNPs is equal to the linear contribution, satisfying *E*_c_ = *F*_c_*C*_abs_. Furthermore, the absorption cross section *C*_abs_ and the critical laser fluence *F*_c_ of the GNSs with radii of 50, 55, and 60 nm at the same reference temperature and pulse duration of 5 ns are calculated as a function of laser wavelength, as shown in Figure 4b. Here, the absorption cross section *C*_abs_ is inversely proportional to the critical laser fluence *F*_c_. To illustrate the intuitiveness and simplicity of the critical laser fluence *F*_c_ in quantitatively describing the nonlinear PA signal of GNPs, the *p*_total_/*p*_linearity_ ratios of GNPs with different radii as a function of pulsed laser fluence are calculated at laser wavelengths of 550 and 642 nm, respectively, as shown in Figure 4b,c. As observed in Figure 4c, the relative relationship of critical laser fluence is *F*_c_(*r* = 50 nm) < *F*_c_(*r* = 55 nm) < *F*_c_(*r* = 60 nm), while the nonlinearity in the PA signal at different pulsed laser fluences is *p*_total_/*p*_linearity_(*r* = 50 nm) > *p*_total_/*p*_linearity_(*r* = 55 nm) > *p*_total_/*p*_linearity_(*r* = 60 nm). In Figure 4d, the critical laser fluence relative relationship was *F*_c_(*r* = 55 nm) < *F*_c_(*r* = 60 nm) < *F*_c_(*r* = 50 nm), at which wavelength, the nonlinearity in the PA signal at different pulsed laser fluences is *p*_total_/*p*_linearity_(*r* = 55 nm) > *p*_total_/*p*_linearity_(*r* = 60 nm) > *p*_total_/*p*_linearity_(*r* = 50 nm). This result indicates that the law of the critical laser fluence relative relationship corresponding to the nonlinearity in the PA signal is consistent with that in Figure 4c. It is known that, for different GNPs, a smaller critical laser fluence indicates that GNPs are more likely to produce strong nonlinearity at low laser fluence. In other words, the total detected PA signal of GNPs with a smaller critical laser fluence would have a larger nonlinear component proportion at the same laser fluence. Therefore, the relative value of the critical laser fluence *F*_c_ can not only measure the nonlinearity in the PA signal of different GNPs under the same pulsed laser irradiation but also indicate the effect of the laser wavelength on the nonlinear PA signal of GNPs (the laser wavelength range should be around the LSPR wavelength. Otherwise, the linear PA signal excited by the absorption of water to the laser energy will play a dominant role, at which point the nonlinear PA signal cannot be observed^28^).

### Nonlinear PA Response of Au@SiO_2_ Core–Shell Nanoparticles

When GNPs are applied in PA imaging as contrast agents, they are usually coated to improve the properties of GNPs, making them more favorable for biomedical applications. The most common coated material among them is SiO_2_. Pang et al.^27^ found that for GNSs with diameters over 50 nm, the coated SiO_2_ would quench the nonlinear PA signal of GNSs, suggesting that the reason was that the presence of SiO_2_ decreases the temperature elevation of water not enough to cause the nonlinear PA signal. In addition, the effect of interfacial thermal resistance was ignored in their work. The coated SiO_2_ can change the absorption characteristics and heat-transfer characteristics of GNSs, affecting the generation of nonlinear PA signals. In this section, the effects of SiO_2_ thickness and Au–SiO_2_ interfacial thermal resistance on the nonlinear PA response of Au@SiO_2_ core–shell nanoparticles are quantitatively investigated from the perspectives of critical energy and critical laser fluence. Hence, it provides references for the regulation of the nonlinear PA response of GNSs.

**Figure 5:**
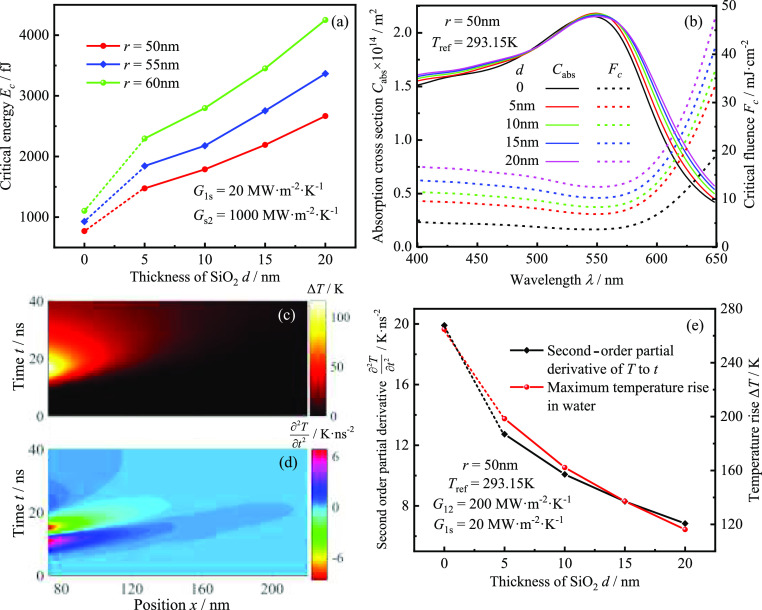
Effect of SiO_2_ thickness on the nonlinear PA response and heat transfer of the Au@SiO_2_ core–shell (reference temperature 293.15 K and pulse duration 5 ns): (a) critical energy of GNSs with different radii as a function of SiO_2_ thickness; (b) absorption cross sections and critical laser fluence of GNSs with different SiO_2_ thicknesses as a function of laser wavelength; (c,d) spatiotemporal distribution of water temperature elevation and ∂^2^*T*/∂*t*^2^ in the radius direction for GNSs with a radius of 50 nm and a laser fluence of 10 mJ/cm^2^, respectively; and (e) maximum temperature elevation and maximum ∂^2^*T*/∂*t*^2^ in water as a function of SiO_2_ thickness.

First, the effect of SiO_2_ shell thickness on the critical energy *E*_c_ of GNSs is investigated, as shown in Figure 5a. It can be seen that the critical energy of GNSs coated with SiO_2_ is significantly larger compared with that of bare GNSs. Meanwhile, the critical energy *E*_c_ increases with the increase of SiO_2_ thickness *d*. However, the change of SiO_2_ thickness will not dramatically affect the absorption cross section of GNSs, as shown in Figure 5b. In contrast, the critical laser fluence of GNSs is increased by the increase of SiO_2_ thickness, indicating that the increase of SiO_2_ thickness will significantly weaken the nonlinear PA response of GNSs. The above phenomenon shows that the SiO_2_ shell can change the heat transfer between GNSs and water. Therefore, it is necessary to analyze the heat-transfer process of the Au@SiO_2_ core–shell in water. It can be seen from Figure 5c that the region of the excitable PA signal is within 200 nm. In the time scale of tens of nanoseconds, the aqueous medium returns to the initial temperature. Meanwhile, the temperature increase of the aqueous medium can reach above 100 K. However, at the nanosecond level time scale and the nanoscale spatial scale, this temperature rise does not generate bubbles, and the generation of nanobubbles usually requires the temperature to reach above 550 K.^42^ However, it is worth noting that the change of water temperature with time is unipolar, while the acoustic wave generated by thermal expansion is bipolar (see Figure 3a), which indicates that the changes between the two are not consistent. In fact, the PA signal excited by the temperature change of water can be described by . Without considering the temperature dependence of the coefficient of thermal expansion, the source term for acoustic pressure is ∂^2^*T*/∂*t*^2^.^41^ The distribution of ∂^2^*T*/∂*t*^2^ with time and location in the radius direction in aqueous medium is obtained from Figure 5c, as shown in Figure 5d. The results show that the profile of ∂^2^*T*/∂*t*^2^ over time is bipolar, consistent with an acoustic pressure signal, and spatially confined to within 160 nm. Moreover, the variation of maximum ∂^2^*T*/∂*t*^2^ versus maximum temperature elevation with SiO_2_ thickness in water is investigated, as shown in Figure 5e. The results show that both the maximum ∂^2^*T*/∂*t*^2^ and the maximum temperature elevation in water decrease with increasing SiO_2_ thickness, which indicates that the increasing SiO_2_ thickness will make the heat-transfer ability of the nanoparticles decrease, and the source term of excitation PA pressure and the thermal expansion coefficient of water simultaneously reduce, making the nonlinearity of nanoparticle PA response weakened.

**Figure 6:**
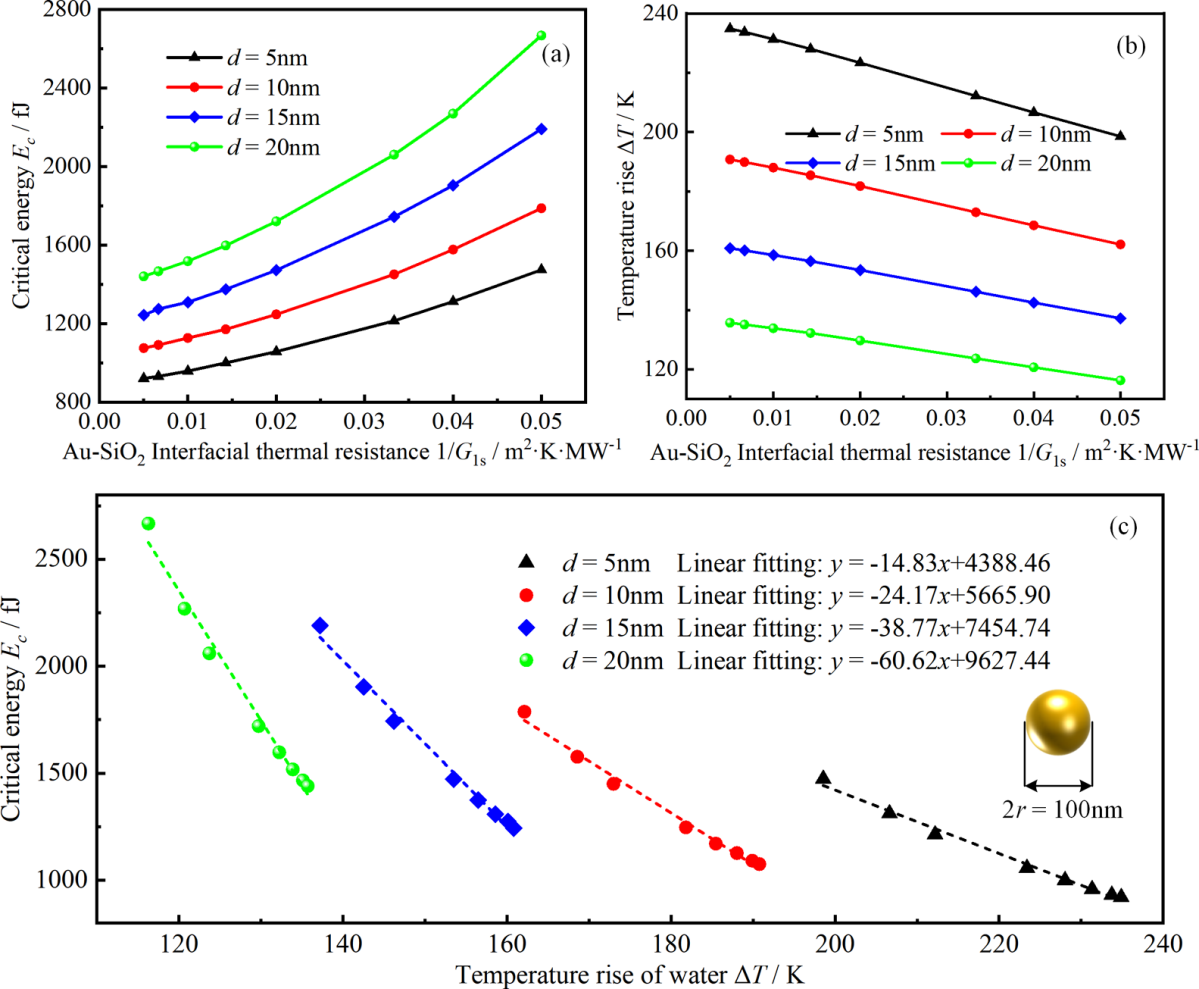
Effect of Au–SiO_2_ interfacial thermal resistance on the nonlinear PA response and heat transfer of the Au@SiO_2_ core–shell (reference temperature 293.15 K, pulse duration 5 ns, and the radius of GNSs 50 nm): (a) critical energy of Au@SiO_2_ with different SiO_2_ thicknesses as a function of Au–SiO_2_ interfacial thermal resistance; (b) maximum temperature elevation in water as a function of Au–SiO_2_ interfacial thermal resistance; and (c) critical energy of Au@SiO_2_ with different SiO_2_ thicknesses as a function of the maximum temperature elevation in the water.

The value of interfacial thermal conductivity *G*_1s_ of Au–SiO_2_ is in the range of 20–200 MW·m^–2^·k^–1^,^41^ which is affected by many factors,^43^ such as wall temperature, curvature radius, and wettability. This parameter has a significant effect on the heat-transfer properties of GNPs under the nanosecond pulsed laser, which in turn affects their PA response. Therefore, it is necessary to investigate the impact of the Au–SiO_2_ interfacial thermal resistance (1/*G*_1s_) on the critical energy and maximum temperature elevation in water, as shown in Figure 6a,b. The results show that the increased thermal resistance of the Au–SiO_2_ interface weakens the heat-transfer ability of GNSs to water, and the maximum temperature elevation of water decreases linearly with increasing Au–SiO_2_ interface thermal resistance, implying that GNSs need to absorb more thermal energy to excite the detectable nonlinear PA signal. Moreover, the critical energy and the maximum temperature elevation of water for the Au–SiO_2_ core–shell are closely related, as shown in Figure 6c. It can be seen that the critical energy is approximately linear with the maximum temperature elevation of water, further illustrating that the nonlinear PA response has a strong dependence on the heat-transfer process.

### Nonlinear PA Response of Gold Nanochains

From the above analysis, we can conclude that SiO_2_-coated GNSs will weaken the nonlinearity of the PA response and that nonlinearity can even disappear when the thickness of the SiO_2_ shell is large. The presence of coated SiO_2_ establishes a direct link with the quenching of nonlinear PA signals of GNSs, which allows one to apply Au@SiO_2_ core–shell nanoparticles as biosensors to monitor some physiological processes.^27^ The nonlinear weakening of the PA response of GNSs by SiO_2_ is achieved by reducing the heat-transfer ability between GNSs and water, the core of which is to reduce the temperature elevation of water. The next question is whether the nonlinear PA response of GNSs can be regulated by controlling the heat-transfer ability between GNSs and water and controlling the temperature elevation of water. In a previous study about the linear PA response of nanoparticles, Chen et al.^44^ made the core–shell ratio of Au@SiO_2_ meet 1:10 to eliminate the plasmonic coupling of nanoparticles. By contrasting the PA signal amplitudes of Au@SiO_2_ core–shell nanoparticles in a dispersed and agglomerated state, it was found that Au@SiO_2_ in the agglomerated state enhanced the heat-transfer ability through the thermal coupling among particles, increased the temperature elevation of water, and enhanced the PA signal. The same phenomenon was also observed by Ju et al.^45^ in the solution of melanin nanoparticles in the agglomerated state. A similar thermal coupling structure also exists for short chains of GNSs, and the thermal coupling effect of the particles can be controlled by controlling the number of nanospheres in the short chains and the gap between the spheres. In addition, the number and gap of nanospheres in gold nanochains can be regulated in a variety of ways, for example, by DNA for assembly.^46−48^ Therefore, in this work, the nonlinear PA response of gold nanochains is investigated.

**Figure 7:**
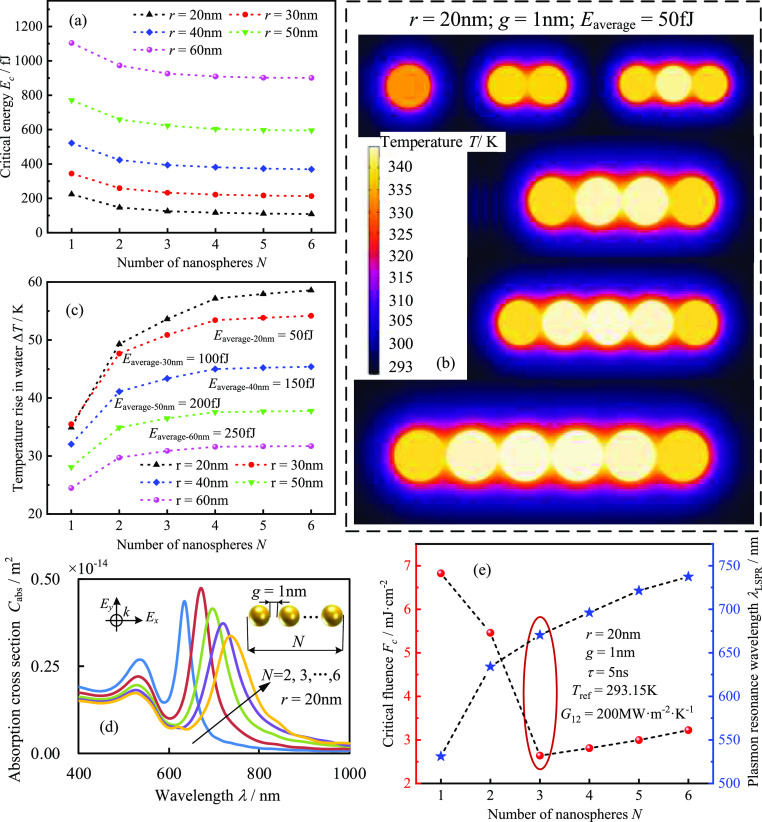
Effect of several nanospheres on the nonlinear PA response and heat transfer of gold nanochains (reference temperature 293.15 K, pulse duration 5 ns, and gap 1 nm): (a) average critical energy of nanochains with different radii as a function of number of nanospheres; (b) variation of temperature distribution of nanochains with a number of nanospheres (laser power maximum moment, *t* = 12 ns); (c) maximum temperature elevation in water as a function of number of nanospheres; and (d,e) average absorption cross sections and critical laser fluence of gold nanochains as a function of number of nanospheres.

First, the average critical energy (total critical energy of nanochains/number of nanospheres) of gold nanochains with different radii as a function of the number of GNSs is calculated, as shown in Figure 7a. The results show that increasing the number of nanospheres decreases the average critical energy of nanochains. When the nanosphere changes from one to two, the variation of average critical energy becomes very obvious. When the number of nanoparticles is larger than 4, the variation can be neglected. The above results can be explained by analyzing the heat-transfer process of the nanochains, as shown in Figure 7b,c. Since varying the number of GNSs can affect both the heat-transfer characteristics and the electromagnetic coupling characteristics, to highlight the effect of the heat-transfer characteristics, for nanochains of the same radius, we set the thermal energy generated by each GNS in the nanochain to be the same, which is expressed by *E*_average_. As observed in Figure 7b, the thermal coupling effect of the dimers significantly increases the temperature of GNSs and the nearby water compared with that of the single nanospheres, indicated as enhanced heat transferability between GNSs and water. This result further indicates the nonlinear enhancement of the PA signal, implying that the average critical energy of the nanochains is significantly reduced. Meanwhile, the enhancement of thermal coupling was still effective from dimer to trimer, trimer to tetramer, but it was significantly weakened when the number of nanospheres exceeded 4. This is because the temperature change region of water under nanosecond pulses is limited to a specific spatial range. In a chain-shaped structure, the other GNSs and water in a more distant region from a certain GNS will not be heated by the sphere. Therefore, the temperature of GNSs and water will not be obviously improved, which indicates that the nonlinearity in the PA response of nanochains will not be enhanced, and the average critical energy will tend to be stable. The above analysis is also proved by the results of maximum temperature elevation in water for the different radii of nanochains, as shown in Figure 7c. Keeping the range of values of the maximum temperature elevation in the water close for nanochains of different radii would indicate that the number of nanospheres on the maximum temperature elevation in water can be highlighted. Therefore, in Figure 7c, it is shown that we make individual nanospheres of different radii nanochains possess different thermal energies.

The aggregation of GNSs will excite not only thermal coupling but also plasmonic coupling, which appears as a second absorption peak on the absorption spectrum, as shown in Figure 7d. As the number of nanospheres increases, the second absorption peak red-shifts with a broader spectrum. Meanwhile, the maximum value of the absorption cross section of a single nanosphere appears in the trimeric nanochains. The above results may enable one to regulate the position of absorption peak wavelength by changing the number of nanospheres in the nanochains, providing a feasible strategy for regulating the absorption spectra of nanochains. Combining the results in Figure 7a,d, the critical fluence as a function of the number of nanospheres is obtained, as shown in Figure 7e. It can be seen that the critical fluence of the nanochains is significantly smaller than that of a single nanosphere due to the combined effect of thermal and electromagnetic coupling, implying that the nanochains can produce a more significant nonlinear PA response. Furthermore, we find that the critical fluence of the trimers is the smallest among the nanochains, with a radius of 20 nm and a gap of 1 nm. Therefore, there is an optimum value for the number of nanospheres in gold nanochains for enhancing the nonlinearity of PA response, and an excessive number of nanospheres is not advantageous.

**Figure 8:**
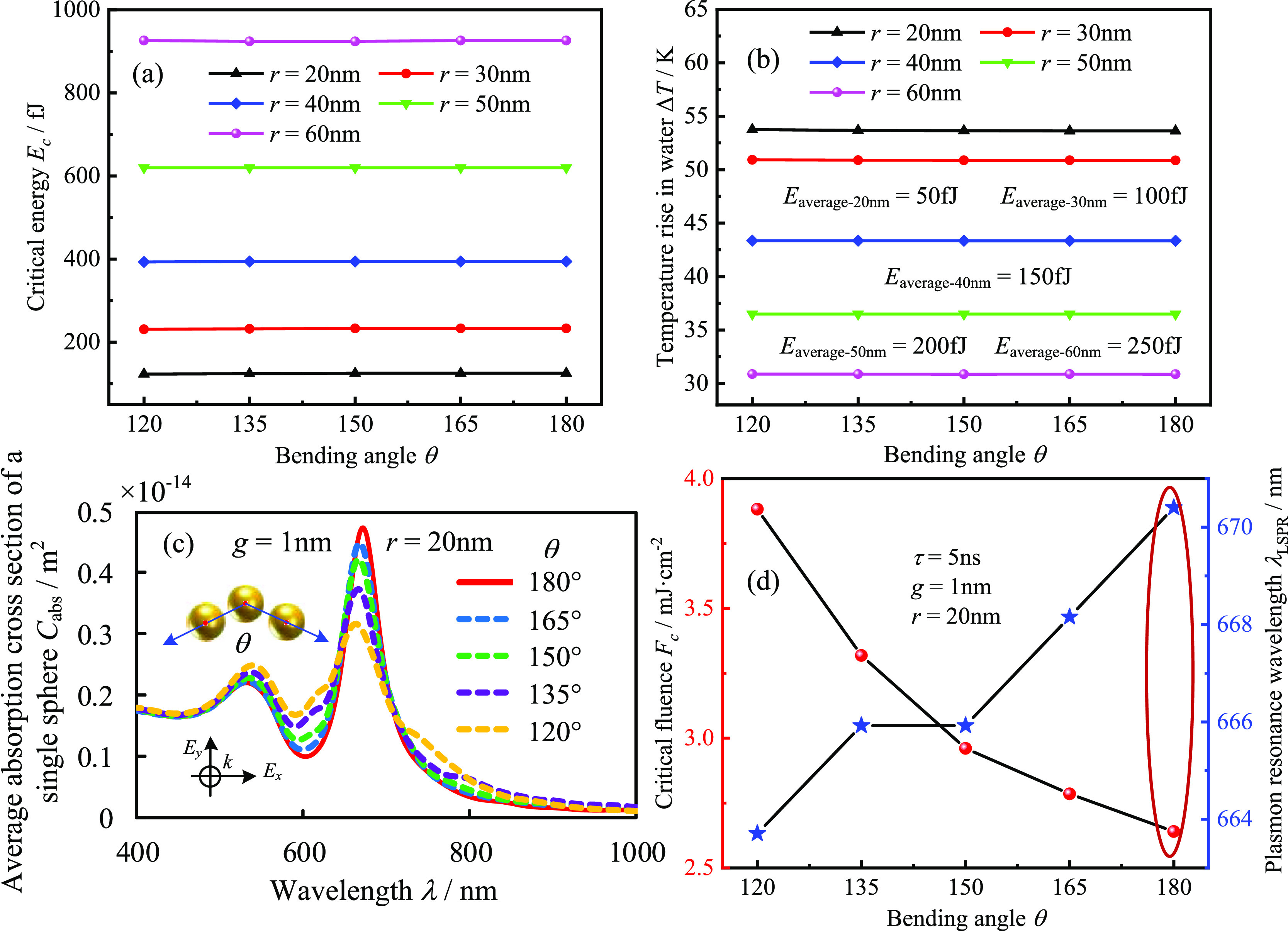
Effect of bending angle on the nonlinear PA response and heat transfer of gold nanotrimers (reference temperature 293.15 K, pulse duration 5 ns, and gap 1 nm): (a) average critical energy of nanochains with different radii as a function of bending angle; (b) maximum temperature elevation in water as a function of bending angle; and (c,d) average absorption cross sections and critical laser fluence of nanotrimers as a function of bending angle.

When the number of nanospheres exceeds 2, it is difficult for GNSs in the nanochains to strictly align along a straight line, and a certain degree of bending must occur. In this work, trimeric nanochains are used as an example to study the influence of bending on the nonlinear PA response of gold nanochains, as shown in Figure 8. As observed in Figure 8a, a certain degree of bending does not affect the critical energy for the trimer because bending does not significantly alter the thermal coupling effect between the GNSs. To illustrate this, set the thermal energy generated by each GNS in the trimer to be the same. However, nanochains of different radii possess different thermal energies, highlighting the effect of bending on heat transfer, as shown in Figure 8b. It can be seen that the maximum temperature elevation of water in the trimer at different bending angles is essentially unchanged. However, bending can affect the electromagnetic coupling of nanochains, manifested by a slight blue shift of the second absorption peak in the absorption spectrum and a decrease in the maximum absorption cross section, as shown in Figure 8c. Furthermore, when the trimeric nanochains are gradually bent, the critical fluence is significantly larger and the nonlinearity in the PA response is weakened due to the significant decrease of the maximum absorption cross section. Therefore, for gold nanochains, bending is not beneficial to improve the nonlinearity in the PA response. Thus, it is best to synthesize linear gold nanochains when applying the nonlinear PA response of nanochains, and a method for the synthesis of ideal linear gold nanochains has been reported.^49^ In addition, in the study of the effect of the number on the nonlinear PA response, we do not consider the effect of bending. However, even if the nanochains are bent, our research results on the number of gold nanochains are still of reference value for the nonlinear PA response of gold nanochains. As indicated, bending does not significantly change the critical energy of the gold nanochains, and the corresponding critical fluence can still be easily obtained through *E*_c_ = *F*_c_*C*_abs_ after the average absorption cross section of the curved gold nanochain is obtained.

**Figure 9:**
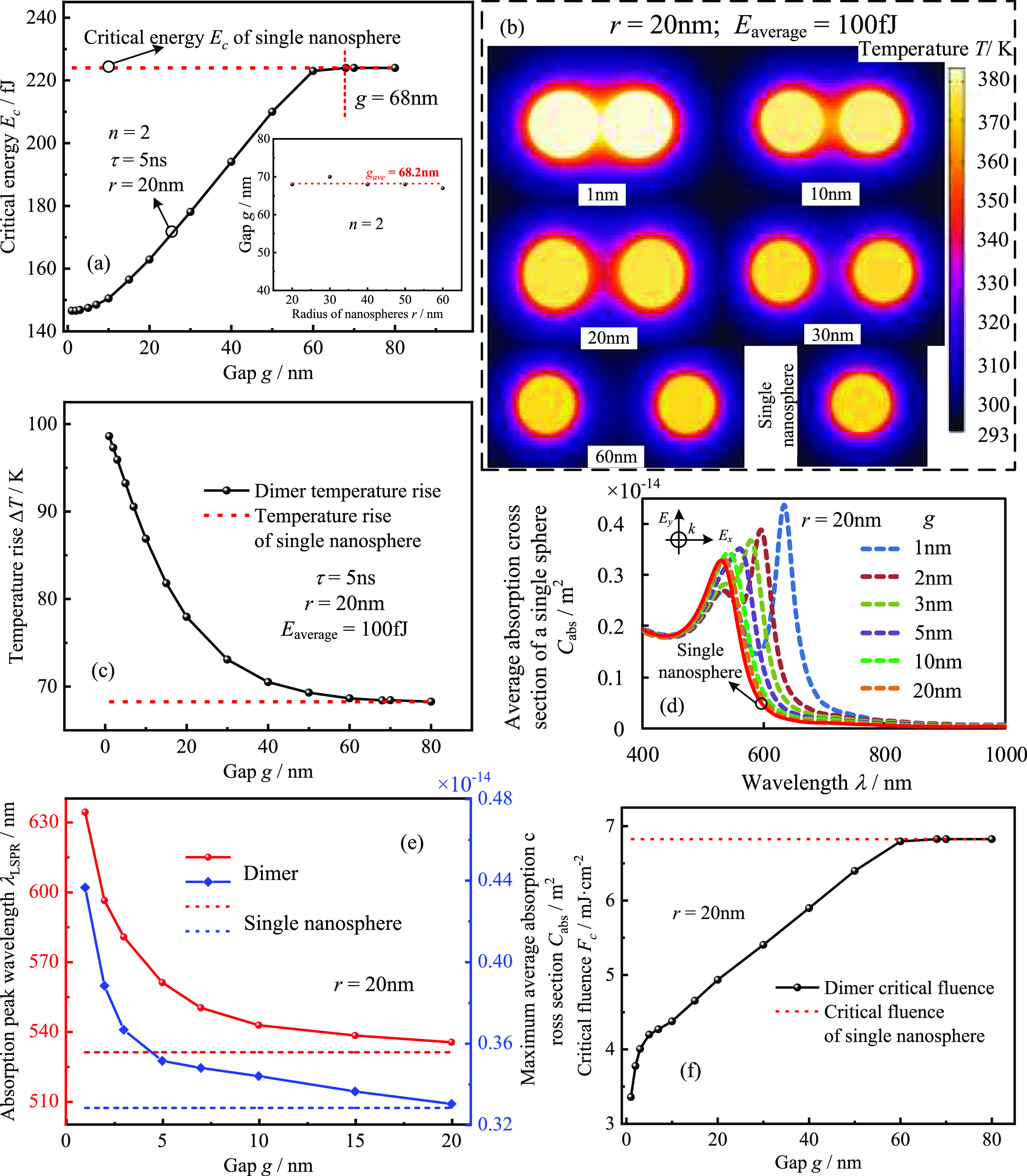
Effect of the gap on the nonlinear PA response and heat transfer of gold nanodimers (reference temperature 293.15 K, pulse duration 5 ns, and the radius of GNSs 20 nm): (a) average critical energy of nanodimers as a function of gap. The critical gap of nanodimers as a function of the radii of nanospheres in the right inset; (b) variation of temperature distribution of nanodimers with the gap (laser power maximum moment, *t* = 12 ns); (c) maximum temperature elevation in water as a function of gap; (d,f) average absorption cross sections and critical laser fluence of gold nanodimers as a function of gap; and (e) absorption peak wavelength and maximum average absorption cross section of nanodimers a function of the gap.

The gap of the nanospheres has an essential effect on both thermal and electromagnetic coupling and, in turn, on the nonlinearity of the PA response. In this work, dimers with a radius of 20 nm are illustrated as an example, as shown in Figure 9. It can be seen from Figure 9a that the average critical energy of the nanochains becomes larger as the gap increases. Meanwhile, after the gap is larger than 10 nm, it has approximately a linear relationship with the average critical energy. In comparison, after the gap reaches 60 nm, the increasing trend of the critical energy slows down significantly. After the gap reaches 68 nm, the average critical energy of the nanochains is the same as that of a single nanosphere, implying that the thermal coupling completely disappears and the two GNSs are independent of each other with no influence on each other. Furthermore, the variation of the critical gap (the gap of the nanospheres when the thermal coupling disappears) with the radius of GNSs is investigated, as shown in the inset on the right side of Figure 9a. Here, the critical gap is close to invariant when the radius of the nanospheres changes, which is different from the electromagnetic coupling between the dimers of GNSs, and the critical gap for the disappearance of the electromagnetic coupling is 2.5 times the diameter of GNSs.^50,51^ The above results can be explained by the thermal coupling between GNSs in which two water sphere thermal domains will heat each other when they are close to each other. Also, under the same pulsed laser, the radius of the water sphere thermal domain (the difference between the radius of temperature change region and the radius of GNSs) depends on the thermal diffusion velocity of water itself, which is a property of water not affected by the size of GNSs. As observed in Figure 9b, the thermal domain overlap of the water sphere gradually decreases with the increasing gap, decreasing water temperature, and weakening thermal coupling. When the gap reaches 60 nm, the overlap part of the thermal domains of the two water spheres is almost negligible, and the temperature distribution of water is virtually the same as that of a single nanosphere. Therefore, in Figure 9a, after the gap reaches 60 nm, the average critical energy of the dimer (*E*_c_ = 223 fJ) has been almost equal to that of a single nanosphere (*E*_c_ = 224 fJ). A similar rule can also be observed from the calculated results of the maximum temperature elevation in water for the dimer, as shown in Figure 9c. At a gap of 60 nm, the maximum temperature elevation in water for the dimer is very close to that of a single nanosphere, while after the gap reaches 68 nm, both become exactly the same.

The increased gap weakens not only the thermal but also the electromagnetic coupling, manifested by the blue shift of the second absorption peak in the absorption spectrum and the decrease of the average absorption cross section at the absorption peak wavelength, as shown in Figure 9d,e. With a gap of less than 5 nm, the gap change will induce a drastic change in the resonance peak wavelength and the average absorption cross section at the resonance peak wavelength. In comparison, after a gap of more than 5 nm, the changing trend slows down gradually. For a GNS dimer with a radius of 20 nm, the critical gap for the electromagnetic coupling to disappear is 100 nm. However, when the gap is 20 nm, the resonance peak wavelength and the maximum average absorption cross section of the dimer have been very close to that of a single nanosphere, as shown in Figure 9e. Therefore, it can be approximated that the effect of the gap on the absorption characteristics of a nanodimer with a radius of 20 nm can be ignored when the gap is larger than 20 nm. Combining the results in Figure 9a,e, the critical fluence of nanodimers as a function of gap can be obtained, as shown in Figure 9f. At gaps of less than 10 nm, the critical energy increase is slow as the gap increases, but the average absorption cross section of the dimer decreases rapidly, implying that the critical fluence becomes rapidly larger at this stage. Therefore, critical fluence change at this gap range is dominated by a weakening of electromagnetic coupling. When the gap is larger than 10 nm, the decrease in the absorption cross section of the dimer slows down significantly as the gap increases, while the critical energy changes approximately linearly with the gap. Therefore, the critical fluence increases approximately linearly with the gap, implying that weakening of thermal coupling mainly determines the change of critical fluence in this gap range. The electromagnetic coupling and thermal coupling are both relatively weak when the gap is larger than 60 nm. The critical fluence of the nanodimer is almost the same as that of a single nanosphere, indicating that the nanodimer will exhibit the same nonlinear PA response as a single nanosphere.

When GNPs are irradiated by a nanosecond pulse laser, the temperature increases rapidly and will transfer heat to water by thermal diffusion, resulting in a huge temperature gradient in water. The so-called boiling under very large temperature gradient is commonly related to the crossing of the spinodal line, which occurs at a temperature (called spinodal temperature 550.15 K and different from the boiling point 373.15 K) just below the critical fluid temperature.^52,53^ It is worth mentioning that although the maximum temperature of the water exceeds the boiling point of 373.15 K, there will be no nanobubbles or gas–liquid phase transition since it is still lower than the spinodal temperature.

## Conclusions

In this work, the dependence of the nonlinear PA response of GNPs on the heat-transfer ability under nanosecond pulsed laser irradiation is investigated using FEM by taking Au@SiO_2_ core–shell nanoparticles and gold nanochains as examples. The nonlinear PA response can be quantitatively analyzed by critical energy. However, the nonlinear PA response of different nanoparticles cannot be directly contrasted by critical energy. We find that the critical fluence can directly represent the proportion of nonlinear components in the PA response of different nanoparticles, that is, the smaller the critical fluence, the higher the nonlinear proportion (or the stronger the nonlinearity) in the PA response. Furthermore, the effect of the heat-transfer ability of GNPs on critical energy is investigated. The results show that the heat-transfer ability is inversely related to critical energy. It is explicitly demonstrated that the increased thickness of coated SiO_2_ and the interfacial thermal resistance of Au–SiO_2_ will make the heat-transfer capacity of GNSs to water decrease. As a result, the maximum temperature elevation in water decreases, thus increasing the critical energy. While the absorption cross section is not significantly changed, the critical fluence increases, and the nonlinearity of PA response is weakened. In contrast, the thermal coupling reduces the critical energy by the enhanced heat-transfer ability of gold nanochains. At the same time, the absorption cross section is significantly increased by the electromagnetic coupling. Therefore, the critical fluence is reduced considerably, resulting in the excitation of a more significant nonlinear PA response. The results about gold nanochains are helpful to understand the mechanism of the effect of aggregation on the nonlinear PA response of GNPs, not just gold nanochains, since the nanochain is a simple and representative aggregation structure in the study of mechanism.

Adjusting the nonlinear PA response by changing the thermal coupling of GNPs has potential applications in biological imaging and biosensors. In fact, the endocytosed GNPs inevitably agglomerate,^54^ while nanoparticles staying near the vascular system will not aggregate significantly.^55^ Due to the difference of thermal coupling, GNPs in different positions (whether in the cell or not) will show significant differences in nonlinear PA response. This provides a new choice for accurate diagnosis of tumors. Moreover, the nanochains formed by coupling GNSs with DNA have a wide range of applications in biosensors.^54^ It has been reported that temperature affects the gap of DNA-bonded nanodimers.^47^ According to our research results, the gap affects the optical properties, which further changes the nonlinear PA response. A new perspective for indirect temperature measurement can be provided through the relationship among temperature, gap, and nonlinear PA response. In addition, Pang et al.^27^ pointed out that due to the great influence of SiO_2_ coating on the nonlinear PA response of GNSs, Au@SiO_2_ nanoparticles can be a potential candidate for biosensors based on nonlinear PA response.
